# Linear modeling of brain activity during selective attention to continuous speech: the critical role of the N1 effect in event-related potentials to acoustic edges

**DOI:** 10.1007/s11571-025-10289-z

**Published:** 2025-07-02

**Authors:** Adrian Mai, Steven A. Hillyard, Daniel J. Strauss

**Affiliations:** 1 Systems Neuroscience and Neurotechnology Unit, Faculty of Medicine, Saarland University & htw saar, Neurocenter, Building 90.5, 66421 Homburg/Saar, Germany; 2 Center for Digital Neurotechnologies Saar, 66421 Homburg/Saar, Germany; 3 Department of Neurosciences, University of California San Diego, La Jolla, CA 92093 USA; 4 Leibniz Institute for Neurobiology, 39118 Magdeburg, Germany

**Keywords:** Event-related potentials, N1 effect, Selective auditory attention, Speech tracking, Stimulus reconstruction, Temporal response functions

## Abstract

Recent studies have suggested a cortical representation of speech through superposition of evoked responses to acoustic edges, an idea closely related to regression-based modeling approaches for studying cortical synchronization to speech via magneto- or electroencephalography (M/EEG). However, it is still unclear to what extent speech-evoked event-related potentials (ERPs) contribute to these techniques. The present study addressed this question by re-analyzing an EEG data set obtained during a selective auditory attention task in which participants focused on one of two competing speakers. Segmenting the EEG based on acoustic edges revealed ERPs with clear P1-N1-P2 complexes and enhanced N1 components elicited by attended streams (*N1 effect*). Comparisons between ERPs and regression results revealed that temporal response functions were highly similar spatiotemporally to the corresponding ERPs and that stimulus reconstruction accuracies were driven by a consistent enhancement of ERPs including the N1 effect. These observations point to a direct link between ERPs to acoustic edges in speech and the linear modeling techniques. In particular, the improvement in signal-to-noise ratio produced by consistent attention-related enhancements of the N1 component was found to be critical for achieving tracking of selectively attended speech, presumably facilitating the higher-order processing of the selected stream.

## Introduction

The investigation of selective auditory attention has a long history in electroencephalographic (EEG) research. Historically, the underlying neural mechanisms have been studied extensively over the last fifty years using event-related potentials (ERPs) (see Picton 2010 for review). In their seminal works, Hillyard and colleagues (Hillyard et al. 1973; Picton and Hillyard 1974) observed that selectively attending to a sequence of brief auditory stimuli in a top-down manner causes an enhancement of the N1 component (having a typical latency of 80-120ms in the auditory ERP compared to the response evoked by an ignored sequence (*N1 effect*). Hillyard et al. (1973) originally interpreted the auditory N1 effect as a gain control operation, such that it would result in an improved signal-to-noise ratio (SNR) between cortical activity related to relevant input compared to unrelated background activity.

While the aforementioned traditional ERP paradigm creates a rather artificial listening scenario, its principal idea can be easily extended to a more naturalistic analog by considering a dual-speaker cocktail party scenario in which a listener may want to focus on one speaker and ignore the other. From a neurophysiological point of view, attending to a speaker’s voice produces modulation of brain activity through synchronization of neural responses to speech features, which can be generalized in a non-specific manner as neural tracking (Zion-Golumbic et al. 2013; Di Liberto et al. 2022). As tracking implies a continuous process, the analysis methods would also require an adaptation to the continuous nature of ongoing speech compared to transient auditory stimuli with well-defined onsets and offsets. In recent years, methods based on linear regression have been rapidly growing in popularity to overcome this obstacle. Supported by the provision of freely available data processing toolboxes (Crosse et al. 2016; Brodbeck et al. 2023), these modeling approaches have led to remarkable insights into the neural tracking of sensory inputs and have been utilized in studies investigating, for example, multi-modal interactions (e.g., Crosse et al. 2015), music perception (e.g., Weineck et al. 2022), pitch processing (e.g., Brodbeck and Simon 2022), linguistic processing (e.g., Gillis et al. 2021), speech intelligibility (e.g., Vanthornhout et al. 2018; Muncke et al. 2022), effects of hearing aid processing strategies (e.g., Alickovic et al. 2020, 2021; Mai et al. 2022), and selective attention (e.g., Schäfer et al. 2018; Teoh et al. 2022). In these regression-based frameworks, continuous relations may be linearly modeled in forward or backward direction. The former approach results in a temporal response function (TRF) representing a characteristic brain response optimized to map a specific stimulus feature to an observed response (see Holdgraf et al. 2017 for review). The latter method implements a stimulus reconstruction (SR) procedure and generates a decoder that can be applied to the observed response to approximate the original stimulus feature. The similarity between this estimation and the actual stimulus representation is then used to quantify the degree of neural tracking (Holdgraf et al. 2017). Studies that have employed these two approaches are reviewed below.

Although forward-modeled TRFs and classical ERPs have mostly been analyzed separately, investigations of the relation between them have revealed some distinct similarities. Throughout the literature, modeled TRFs have closely resembled the morphology of ERPs, as they consisted of short oscillatory waveforms with clear components (Lalor et al. 2009; Lalor and Foxe 2010; Power et al. 2012; Crosse et al. 2015; Di Liberto et al. 2015; Fiedler et al. 2019; Drennan and Lalor 2019; Lesenfants and Francart 2020; Muncke et al. 2022; Weineck et al. 2022). Furthermore, studies of selective auditory attention have consistently reported waveform modulations, mainly in the N1 latency range, for TRFs fitted to attended and ignored auditory streams, in line with the SNR-enhancing auditory N1 effect (Ding and Simon 2012a, b; Fiedler et al. 2017, 2019; Kaufman and Zion-Golumbic 2023). Along these lines, it was also shown that an increased SNR between tracking- and non-tracking-related activity was important for obtaining accurate TRF estimations (Crosse et al. 2021). Because TRFs and the corresponding SR decoders are mathematically related (Haufe et al. 2014), it would be expected that the N1 effect and the resulting SNR enhancement would translate to both forward and backward modeling approaches. Indeed, a consistent outcome of SR analyses in selective attention studies has been reliably higher reconstruction accuracies for attended compared to ignored auditory streams (O’Sullivan et al. 2015; Fuglsang et al. 2017; Puschmann et al. 2017; Hausfeld et al. 2018; Schäfer et al. 2018; Wong et al. 2018; O’Sullivan et al. 2019; Alickovic et al. 2020; Teoh et al. 2022; Mai et al. 2022).

An important finding that may provide a link between continuous and event-related approaches to speech analysis was recently made by Oganian et al. (2023). By segmenting ongoing magnetoencephalographic (MEG) data based on acoustic edges in speech as represented by salient intensity dynamics, they identified event-related activity that closely resembled the P1-N1-P2 complex of the auditory ERP. It seems reasonable to assume that a similar effect may be present in EEG recordings and that the N1 component of the speech-evoked ERP in multi-speaker scenarios would exhibit an N1 enhancement analogous to that observed in auditory ERPs to simple tone pips or clicks (Hillyard et al. 1973; Picton and Hillyard 1974). If so, a consistent SNR improvement in favor of an attended speaker compared to any distracting sources produced by enhanced N1 components in speech-evoked ERPs to acoustic edges may facilitate the neural tracking of the attended speech. In addition, it should be noted that the dominant spectral content of the N1 component is located within the theta range (4-8Hz) (Klimesch et al. 2004; Trenado et al. 2009; Low and Strauss 2011; Bernarding et al. 2017; Corona-Strauss and Strauss 2017), a frequency band known to be significantly involved in the neural synchronization to speech (Luo and Poeppel 2007; Kerlin et al. 2010; Giraud and Poeppel 2012; Di Liberto et al. 2015; Chalas et al. 2023).

**Fig. 1:**
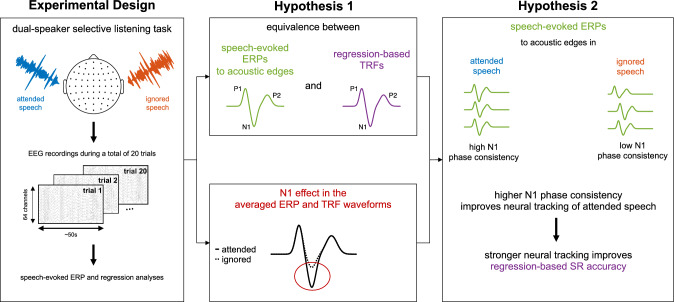
Summary of the experimental design and our working hypotheses. Participants engaged in a dual-speaker selective auditory attention task in which they had to focus on one of two competing speakers while multi-channel EEG recordings were obtained. Continuous speech stimuli were presented via insert earphones, perceived at $\pm{60}^{\circ }$ from the midline, and the to-be-attended location was randomly assigned across 20 trials (each lasting 50s). Our first hypothesis was that speech-evoked ERPs and modeled TRFs would be highly correlated if both are obtained from the same stimulus representation, including similar effects of selective auditory attention with enhanced mean N1 amplitudes in responses elicited by the attended speech. The second main hypothesis was that SR performance would be better for the attended speech due to highly phase-locked N1 responses elicited by acoustic edges, compared to the inconsistent N1 responses with low SNR elicited by the ignored speech. This would produce a more accurate neural representation of the attended speech that could be interpreted to reflect a sustained attention-driven SNR enhancement in favor of the attended stream.

Considering the evidence discussed above, we propose that the observed results in regression-based analyses of selective speech tracking may be attributed in large measure to the generation of speech-evoked ERPs including the well-established N1 effect and the accompanying SNR improvement. In order to address this question, we re-analyzed a published EEG data set (Fuglsang et al. 2018) that was previously used to study speech tracking in deteriorated acoustic scenes (Fuglsang et al. 2017) and the effects of different regularization techniques on forward and backward model estimations (Wong et al. 2018). These EEG data were obtained in a selective attention task in which participants were cued on each trial (lasting 50s) to attend to one of two concurrent speakers perceived at azimuth angles of $\pm{60}^{\circ }$. Our analyses were based on the premise that speech-evoked ERPs may be extracted in response to salient intensity dynamics of the speech envelope, as has been demonstrated using MEG recordings (Oganian et al. 2023). Given the close conceptual relationship between the proposed mechanism of repeated ERPs to speech and the linear forward modeling approach, we first hypothesized a direct correspondence between speech-evoked ERPs and modeled TRFs, with similar effects of selective auditory attention manifested through the N1 effect. Our second hypothesis was that the consistency of the attention effect on speech-evoked ERPs as quantified by the stability of the instantaneous ERP phase in the theta band, which could be interpreted as a measure of sustained SNR enhancement, would correlate with the decoding performance of the SR approach. These working hypotheses are illustrated in schematic form in Fig. 1. Indeed, the present results suggest a fundamental relation between top-down modulation of ERPs to salient changes in speech dynamics and linear regression analyses utilizing the corresponding speech feature representation.

## Materials and methods

### Data availability statement

The analyzed data set was created within the *Cognitive Control of a Hearing Aid* (COCOHA) project and is freely and publicly available (Fuglsang et al. 2018). Sections2.5*Participants*, *Auditory stimuli*, *Experimental procedure*, and *Data acquisition* provide a summary of the information given in the associated publications (Fuglsang et al. 2017, 2018; Wong et al. 2018). Starting with Section *EEG preprocessing*, we report the additional processing that we applied to the raw data set in its distributed format for the present analyses.

### Participants

While the original study comprised 29 participants (13 females, 4 left-handed) aged between 19 and 30 years with normal hearing and no neurological disorder history as stated via self-report (Fuglsang et al. 2017), the available data set includes a subset of 18 subjects (Fuglsang et al. 2018). Participants were required to sign an informed consent form following the regulations of the Declaration of Helsinki and were financially compensated for their voluntary participation. The study design was approved by the Science Ethics Committee for the Capital Region of Denmark.

### Auditory stimuli

Auditory stimuli were fictional stories in the Danish language that were narrated by two professional speakers (male and female) and segmented into consecutive epochs of 50s. All speech recordings were performed in an anechoic chamber at the Technical University of Denmark, and stimuli were provided with a sampling frequency of 44.1kHz. As the purpose of the original publication was to investigate cortical tracking of speech in real-world scenarios (Fuglsang et al. 2017), stimuli were modified to mimic different degrees of reverberation as well as different speaker positions. Different perceived speaker positions were achieved by convolving the speech stimuli with impulse responses derived from non-individualized head-related transfer functions (HRTF) for azimuth angles of $\pm{60}^{\circ }$, an elevation of ${0}^{\circ }$, and a source distance of 2.4m. HRTFs were obtained from simulated auditory scenes (ODEON V13.02, Odeon A/S, Denmark) with either anechoic properties or low (${\sim}189{m}^{3}$ room volume) and high (${\sim}{39000}{m}^{3}$ room volume) degrees of reverberation, resulting in three acoustic conditions. The original experiment consisted of 70 trials with 20 trials for each of these three conditions and 10 trials of an additional anechoic single-speaker scenario in which only the male speaker was presented. Since the aim of the present study was to investigate pure selective attention effects without any influences from acoustic degradation, all further analyses were based on the 20 anechoic dual-speaker trials. Speech stimuli were presented via insert earphones (ER-2, Etymotic Research, Inc., USA) at an intensity level of 65dB SPL and normalized to have the same root-mean-square amplitude in all dual-speaker scenarios.

### Experimental procedure

Since the experimental procedure differed between two participant groups, we refer to the original publication (Fuglsang et al. 2017) for all details and only report the procedure for the data set included here. In each experimental trial, a dual-speaker scenario was created by simultaneously presenting a single speech segment from one speaker perceived at $+{60}^{\circ }$ and the competitor at $-{60}^{\circ }$. Participants had to engage in a selective attention task by attending to the speech stream they were cued to before trial onset while focusing their gaze on a fixation cross and reducing movements as much as possible. A subsequent analysis of comprehension questions related to the content of the target story, which were asked after each trial, validated the subjects’ compliance (Fuglsang et al. 2017). To avoid any undesired systematic biases due to the experimental procedure, the order of the acoustic conditions, the gender and position of the target stream as well as the presentation order of the stories were randomized across trials. All recordings took place in an electrically shielded and soundproof room.

### Data acquisition

EEG acquisition was coordinated by a biopotential recording system (ActiveTwo, BioSemi, The Netherlands) with a 64-channel cap configured according to the international 10-20 system. Additional electrophysiological recordings included the signals from the left and right mastoids as well as vertical and horizontal electrooculograms for both eyes. All data were digitized at 512Hz along with trigger signals indexing the onset and offset of each trial within each participant’s measurement session.

### EEG preprocessing

Data processing was implemented in MATLAB^©^ (R2022a, The MathWorks, Inc., USA), and all reported filtering procedures were conducted via forward and backward passes with 3rd order Butterworth filters providing 3dB attenuation at the cutoff frequencies. Raw EEG data were decimated to 256Hz and bandpass-filtered from 1-45Hz. Noisy channels were removed based on their time courses and power spectra in EEGLAB (V2022.0, Delorme and Makeig 2004), and the remaining EEG channels were re-referenced to the average of the mastoids. Following an independent component analysis (ICA) decomposition of the EEG data, artifactual components were removed after visual inspection informed by the ICLabel plugin (Pion-Tonachini et al. 2019), and the data were back-projected to sensor-space. ICA decomposition was achieved via the AMICA algorithm (Palmer et al. 2008, 2012), which has been shown to outperform other blind source separation techniques in maximizing near-dipolarity while minimizing mutual information of independent components (Delorme et al. 2012). Previously discarded EEG channels were finally interpolated using EEGLAB’s default routine for spherical interpolation to obtain complete data sets, and all channels were corrected for their DC-offset.

### Speech envelope processing

The subsequent EEG analyses carried out in our laboratory were based on envelope representations of speech stimuli. Envelope extraction was carried out following similar procedures to those previously implemented by Oganian and colleagues (Oganian and Chang 2019; Oganian et al. 2023), but was adapted to include signal transformations commonly applied in electrophysiological speech tracking analyses. Raw speech waveforms were decomposed by a gammatone filter bank (Patterson et al. 1992; Slaney 1993) into 128 subbands with center frequencies between 100Hz and 8000Hz. Individually extracted narrowband Hilbert envelopes were power-law-transformed ($x^{0.3}$) to simulate loudness perception within the auditory system (Stevens 1955) and subsequently averaged to obtain the broadband envelope. To emphasize salient dynamics, broadband envelopes were converted to speech onset envelopes via lowpass filtering at 25Hz followed by differentiation and halfwave rectification (Hambrook and Tata 2014). The resulting train of gaussian-like pulses was expected to correlate with acoustic edges in speech, and consequently to provide appropriate markers for segmentation of ongoing EEG into transient speech-evoked responses.

### Speech-evoked ERP extraction

**Fig. 2:**
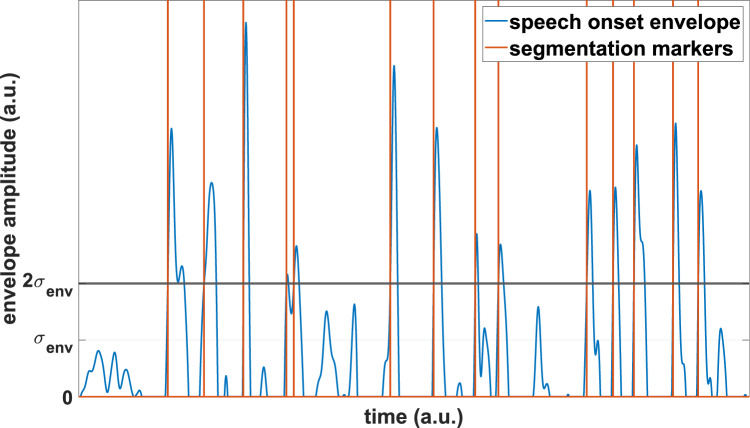
Extraction of segmentation markers from speech onset envelopes for speech-evoked ERPs. Triggers were inserted at each point in time where a gaussian-like pulse in the speech onset envelope first exceeded a pre-defined threshold. The threshold $2\sigma _{env}$ was chosen as twice the standard deviation across all pooled envelope samples within a participant

Speech-evoked ERPs were obtained for each participant, EEG channel, and for the attended and the ignored condition. Preprocessed EEG data were lowpass-filtered at 30Hz, and speech onset envelopes were decimated to 256Hz. To exclude any edge artifacts from filtering, the first and last second of data in each trial were discarded. The subsequent definition of EEG segmentation markers was based on the statistical properties of intensity dynamics in speech envelopes and extended the method of Oganian et al. (2023) by introducing a segmentation threshold. In particular, the corresponding 40 speech onset envelopes were pooled within participants (2 envelopes per trial and 20 trials in total), the global standard deviation $\sigma _{env}$ was calculated across all envelope amplitude values, and a threshold was set at $2\sigma _{env}$. All envelopes were subsequently converted into trigger sequences by inserting segmentation markers at all time instances at which the envelopes changed from sub- to suprathreshold amplitudes as illustrated in Fig. 2. ERPs were then extracted over the interval $-$500ms to 2000ms relative to trigger onsets, and data were pooled across all trials. Following a channel-wise baseline correction by subtracting the mean amplitude between $-$50ms and 0ms, sweeps in which any of the channels exceeded absolute amplitudes of 100$\upmu$V were identified as artifacts and excluded from analysis. This procedure yielded a minimum of 2614 responses for each subject to both attended and ignored stimuli, corresponding to approximately 2.7 triggers per second. Accordingly, 2614 artifact-free ERPs were randomly selected per condition for each participant and submitted to further analysis. Comparisons of ERPs and TRFs were carried out over the time interval from $-$50ms to 500ms.

### Speech-evoked ERP consistency analysis

To examine the consistency of speech-evoked ERPs, instantaneous phase (IP) information was extracted from analytic continuous wavelet transforms using generalized Morse wavelets $\psi _{\gamma , \beta }$ (Lilly and Olhede 2009). As opposed to the commonly applied Morlet wavelet, Morse wavelets provide perfect analyticity (i.e., no support at negative frequencies); a desirable property for obtaining accurate transform coefficients (Lilly and Olhede 2009). All selected sweeps were processed with wavelets from the dual-parameter family $\psi _{3, 3.29}$ to maximize symmetry in time and frequency with an almost minimal Heisenberg area and one full oscillation cycle at the wavelet peak frequency within the central energy window. ERPs were analyzed within the time frame of $-$500ms to 2000ms with respect to the speech onset envelope segmentation markers with 17 scales per octave within 2-32Hz. The extracted IP angles were subsequently used to compute the wavelet phase synchronization stability (WPSS; also called phase-locking factor (Tallon-Baudry et al. 1996) or inter-trial phase-coherence (van Diepen and Mazaheri 2018)), which provides a measure of morphologic waveform consistency across a set of responses; this measure has been employed in different forms for various analyses within the auditory domain including listening effort (Strauss et al. 2010; Bernarding et al. 2013; Wisniewski 2017), selective attention (Low and Strauss 2011; Fuglsang et al. 2020), and tinnitus (de-)compensation (Strauss et al. 2008; Haab et al. 2019). Specifically, the WPSS at a particular wavelet scale and translation was quantified as the mean resultant vector length of a set of unit vectors oriented according to the corresponding IP angles at that time-frequency point across all single sweeps, with values bounded between 0 and 1 for perfect desynchronization and synchronization, respectively. The ERP WPSS matrices were computed for each participant, EEG channel, and condition, and trimmed to span the range from $-$50ms to 500ms.

Although similar methods have been applied to investigate the generative processes of ERPs, i.e., phase-reset vs. additive mechanisms (Makeig et al. 2002; Mishra et al. 2012), it is questionable whether these methods provide unequivocal evidence for one or the other process (Yeung et al. 2004; Burgess 2012). Therefore, for the present purposes, the WPSS is considered only as a mathematical tool to quantify response consistency without implying any evidence regarding the generative processes of speech-evoked ERPs.

### Encoding and decoding preprocessing

Linear modeling procedures were carried out using the same EEG data and speech onset envelopes that were used for ERP segmentation (see filtering, decimation, and trimming procedures in Section *Speech-evoked ERP extraction*), and each EEG channel was additionally centered around 0$\upmu$V. A subject-dependent normalization was applied to the EEG data by dividing all amplitude values by the global standard deviation across all channels and trials. Decimated envelopes were normalized within participants by dividing all amplitude values by the global standard deviation across all conditions and trials.

### Encoding/forward modeling

Neurophysiological forward models of speech tracking interpret the neural response *r* at a single channel as the sum of a convolution between a continuous speech feature representation *s* and a TRF *h* which represents a channel-specific stereotypical impulse response, and an unexplained noise component $\epsilon$. By considering observations at integer multiples of the sampling period $t = t_{1}, t_{2}, \ldots , t_{T}$, multiple data channels with indices $n = 1, 2, \ldots , N$, and a range of time lags $\tau$ between $\tau _{min}$ and $\tau _{max}$ relative to *t* with $\tau \in \mathbb {R}^{1\times L}$, the forward model can be expressed as

$$\begin{aligned} r(t,n) = \sum _{l = 1}^{L} s(t-\tau _{l}) h(\tau _{l},n) + \epsilon (t,n) \end{aligned}$$

which evaluates how the stimulus is encoded in the neural activity at channel *n* and time *t* (Holdgraf et al. 2017). Commonly, TRFs are identified via optimization procedures to minimize the mean-squared-error between the actual and predicted neural responses. Ignoring the constant term of the linear model and introducing a compact matrix notation with the multi-channel data set ${\textbf {r}} \in \mathbb {R}^{T\times N}$, a matrix of concatenated, lagged versions of a single-channel stimulus feature ${\textbf {S}} \in \mathbb {R}^{T\times L}$, the identity matrix ${\textbf {I}} \in \mathbb {R}^{L\times L}$, and a regularization scalar $\lambda$ to penalize large filter weights and thereby mitigate overfitting, the multi-channel solution ${\textbf {h}} \in \mathbb {R}^{L\times N}$ can be efficiently obtained via ridge regression,

$$\begin{aligned} {\textbf {h}} = ({\textbf {S}}^{T} {\textbf {S}} + \lambda {\textbf {I}})^{-1} {\textbf {S}}^{T} {\textbf {r}} \end{aligned}$$

which resembles a regularized version of an optimal Wiener filter (Wiener 1964). Instead of estimating the model parameters using ridge regression, the weights can also be obtained using different regularization techniques, e.g., low-rank approximation, shrinkage, Tikhonov regularization, or elastic net regression (Wong et al. 2018). Since these methods have been found to perform similarly well (Wong et al. 2018) and the ridge regression is commonly chosen as the default (Crosse et al. 2016), it was also used for all modeling procedures in the present study. Encoding models were fitted in a subject-dependent manner for each EEG channel including time lags from $-$250ms to 700ms and incorporating data from all 20 trials simultaneously. The regularization included 20 logarithmically spaced parameters $\lambda$ within a broad range of $10^{-6}$ to $10^{6}$ similar to those used or proposed in previous works (Crosse et al. 2016; Wong et al. 2018; Crosse et al. 2021). However, instead of performing a cross-validation procedure to optimize parameter selection, TRFs were separately trained on all 20 values, and the resulting filter weights were averaged. The incorporation of different levels of smoothing may not have fully maximized modeling accuracies to the same extent as, for example, a group-level parameter optimization (Alickovic et al. 2020, 2021), but the main priority was put on a different aspect. In particular, the implemented approach stabilized the regularization across participants and conditions to maximize consistency while reducing impacts of single parameters that could perform especially well or badly for some data sets and keeping the complexity of TRF modeling to a reasonable degree. Repeating the procedure for speech onset envelopes of attended and ignored streams, this resulted in a single model per participant, EEG channel, and condition. TRFs were finally filtered with a 30Hz lowpass to remove ringing artifacts and baseline-corrected analogously to the ERPs. For the following illustrations and comparisons to speech-evoked ERPs, the 200ms buffers at both edges were excluded so as to have matching epochs for TRFs and ERPs.

### Decoding/backward modeling

Neurophysiological backward models of speech tracking follow the same framework as the forward models but include a change in the dependent and the independent variables. These models aim to reconstruct an estimation $\hat{s}$ of an original stimulus feature using a spatiotemporal decoding filter *g*, as given by

$$\begin{aligned} \hat{s}(t) = \sum _{n = 1}^{N} \sum _{l = 1}^{L} r(t+\tau _{l},n) g(\tau _{l},n) \end{aligned}$$

which highlights the possibility of a multi-variate SR approach via integration of information across multiple channels as compared to the univariate forward modeling. The ridge regression solution can again be obtained via the reverse correlation technique

$$\begin{aligned} {\textbf {g}} = ({\textbf {R}}^{T} {\textbf {R}} + \lambda {\textbf {I}})^{-1} {\textbf {R}}^{T} {\textbf {s}} \end{aligned}$$

but now includes a time-lagged version of the neural data instead of the stimulus (Holdgraf et al. 2017). Decoding models were fitted in a subject-dependent manner on a training set of 19 trials including time lags from 0-500ms and with the same regularization approach as for the encoding models. The resulting 19 decoders were averaged and applied to the EEG data of the held-back test trial to obtain an estimation of the corresponding speech onset envelope. Afterwards, SR accuracy was assessed by calculating Pearson’s correlation *r* between the original and the estimated envelope. The leave-one-out procedure was repeated until each trial was labeled as test set once, and models were separately trained and tested for attended and ignored speech. Finally, SR accuracies within participants were averaged across trials. The backward modeling procedure was conducted for each EEG channel individually to allow channel-wise analysis of speech tracking.

### Auditory Attention Decoding

The straightforward selective attention task of the present study provides an excellent opportunity to compare different measures for their ability to decode auditory attention to speech as well as to investigate whether they exhibit similar patterns across EEG channels. In selective speech tracking studies, the effect of attention has commonly been studied via the SR approach (see Introduction). According to the hypotheses of this study (see Fig. 1), SR was expected to be driven by a consistent generation of speech-evoked ERPs, which would include an N1 response enhanced by attention in line with the auditory N1 effect. Translating this idea to the present analysis methods, this should be reflected in a comparably stronger ERP WPSS within the N1 time-frequency area for ERPs to attended relative to ignored speech. It is known that the theta band, a critical frequency range in speech tracking studies, encompasses the dominant spectral content of the N1 component and exhibits WPSS maxima around N1 peak latencies (see Introduction). Due to the nature of the wavelet transform, it follows that this phenomenon would be dominantly captured by analytic wavelets centered at the N1 peak with center frequencies within the theta frequency range. Assuming a minimum of one cycle of the center frequency within the central wavelet energy window to achieve a reasonable time-frequency trade-off as implemented in the present case, this would result in wavelet footprints of at least 125-250ms within 4-8Hz and consequently, a bidirectional energy spread at the center frequency of approximately 62.5-125ms around the N1 peak. This spreading prevents a perfect differentiation between pure N1 contributions and influences from neighboring components such as the P1 and P2. Therefore, the present wavelet analysis considered information within a broader time range based on the oscillatory behavior of ERPs. While not making any assumptions about the interdependence of adjacent ERP components, the P1-N1-P2 complex can be interpreted as being composed of a P1-N1 and a N1-P2 half-cycle with different degrees of theta and alpha contributions. The P1-N1 complex consists mainly of activity in the alpha band (Klimesch et al. 2004), which is consistent with the speech-evoked ERPs to attended speech at channel Cz shown in Fig. 4A, as the P1-N1 half-cycle represents an oscillation of 9.1Hz. In contrast, the N1-P2 complex correlated with an oscillation of 5.6Hz, well within the theta band. Due to its significant involvement in cortical speech tracking and to obtain a robust WPSS attention decoding measure, the ERP WPSS maps were reduced to scalar values by averaging within the theta band from 4-8Hz and in the time window of the N1-P2 complex. Specifically, the time window was chosen according to the peak latencies of ERPs to attended speech at channel Cz with a 20ms buffer before and after the N1 and P2 peaks, respectively. With peak latencies of 136.7ms (N1) and 226.6ms (P2), this resulted in a time frame of 116.7-246.6ms. In order to be consistent with this WPSS methodology, attention decoding measures from ERPs and TRFs were based on N1-P2 amplitudes. N1 and P2 amplitudes were identified as the mean voltage within time windows with centers chosen again according to the peak latencies of these components in response to attended speech at channel Cz. This procedure resulted in time windows 136.7$\pm$20ms and 226.6$\pm$20ms for ERPs and 125.0$\pm$20ms and 214.8$\pm$20ms for TRFs for the N1 and P2, respectively (see Fig. 4A/C). N1-P2 amplitudes were finally defined as the difference between P2 and N1 amplitudes. All decoding measures were computed for each participant, EEG channel, and condition.

### Statistical analyses

Statistical contrasts between the attended and the ignored condition were performed with non-parametric permutation tests (Holmes et al. 1996; Nichols and Holmes 2001; Maris and Oostenveld 2007) for waveforms and ERP WPSS matrices, and with paired *t*-tests for correlations and attention decoding measures. All *t*-tests were conducted one-tailed as it was expected that attention would have an enhancing effect on all measures. Non-parametric permutation tests were performed using within-subject averages as the unit of observations, two-tailed paired *t*-tests as test statistic, and 10000 permutations. A cluster-mass-based approach was applied to correct for multiple comparisons within channels, with pre-clustering and cluster-level thresholds of 0.01 and 0.05, respectively.

## Results

### Speech-evoked ERPs and comparison to TRFs

**Fig. 3:**
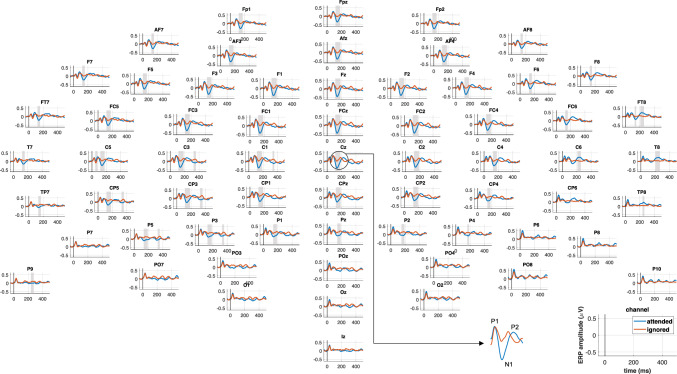
Topographic overview of speech-evoked ERPs. Grand-average ERPs across all participants to attended and ignored speech are represented by solid blue and orange lines, respectively. Grey shadings indicate periods with significant differences between conditions as determined by a non-parametric permutation test. The majority of channels show similar ERP morphologies with components especially pronounced over frontocentral scalp regions. The principal effect of attention was a substantial amplitude increase in the N1 component.

**Fig. 4:**
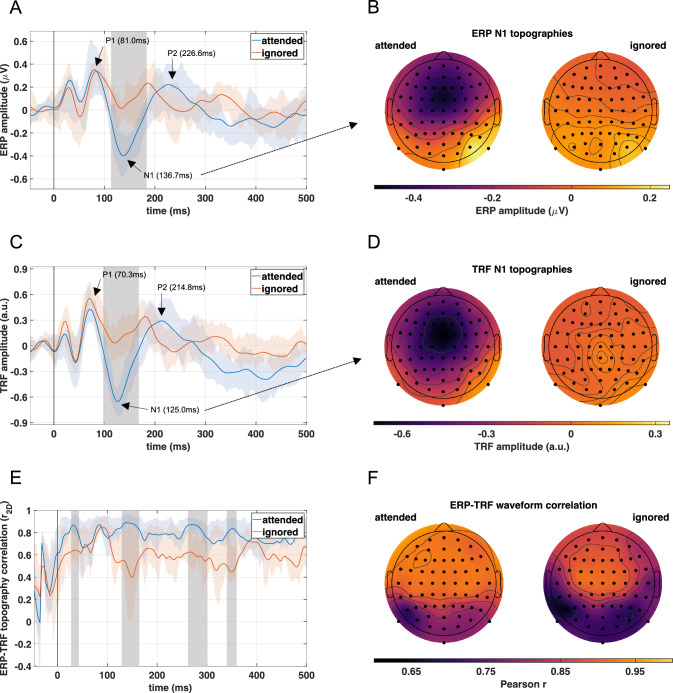
Comparison between speech-evoked ERPs and TRFs resulting from forward modeling. Grand-average ERPs (**A**) and TRFs (**C**) across all participants at channel Cz to attended and ignored speech are represented by solid blue and orange lines, respectively. Colored shadings indicate the interquartile range across participants. Grey shadings highlight periods during which a non-parametric permutation test revealed significant differences between conditions. ERPs as well as TRFs exhibited a significant N1 effect with greater amplitudes in responses to attended speech. The corresponding N1 topographies of the ERPs (**B**) and TRFs (**D**) at their peak latencies (136.7ms and 125.0ms, respectively) both demonstrate a frontocentral distribution for responses to attended speech and an absence of any apparent spatial pattern for ignored speech. A spatial correlation analysis between corresponding ERPs and lag-corrected TRFs (**E**; layout identical to panels **A** and **C**) revealed that the topographies overall showed high similarities for both conditions. However, there was a significantly greater topographic correlation between ERPs and TRFs to attended speech around the N1 peak latency as well as during earlier and later time intervals that were not analyzed further. Correlations between grand-average ERPs and lag-corrected TRF waveforms at individual scalp sites (**F**) revealed that the similarity across channels was significantly higher between ERPs and TRFs to attended versus ignored speech.

A topographic display of the grand-average ERPs across participants to attended and ignored speech is shown in Fig. 3. While the majority of EEG channels showed similar waveforms with distinct ERP deflections including the P1-N1-P2 complex, the components were most pronounced at frontocentral scalp locations. Focusing on the Cz channel shown in Fig. 4A, the ERPs for both conditions exhibited similar morphologies up to the P1 deflection between 75-85ms, with an earlier middle latency component at about 30ms. The P1 component was followed by diverging later responses, with substantially higher amplitude deflections for ERPs to attended speech for several components, including the N1 peaking at 136.7ms, P2 at 226.6ms, as well as a later negativity (>300ms). A non-parametric permutation test confirmed that the effect of attention on the N1 was significant (see Figs. 3, 4A). Additionally, the scalp topographies at the N1 peak latency shown in Fig. 4B demonstrated a broad frontocentral distribution for ERPs to attended speech and no apparent pattern for the ignored condition.

The grand-average TRFs at channel Cz across participants resulting from forward modeling to attended and ignored speech are shown in Fig. 4C. Like the corresponding ERPs shown in Fig. 4A, waveforms for both conditions initially followed a similar course with an earlier component at around 20ms and a P1 between 65-75ms. Again, the N1-P2 complex was noticeably more pronounced in the TRFs to attended speech but exhibited slightly earlier peak latencies than their ERP equivalents, with N1 peaking at 125.0ms and P2 at 214.8ms. Nevertheless, the TRF topographies at the N1 peak latency for attended speech shown in Fig. 4D were nearly identical to the corresponding ERP topographies, presenting a distinct frontocentral distribution for the attended and no apparent pattern for the ignored condition. A non-parametric permutation test again indicated a prolonged significant effect of attention around the N1 peak latency in the TRFs.

To quantify the similarity between ERPs and TRFs (see working hypothesis 1 in Fig. 1), two types of correlation analyses were carried out. Since TRF peaks consistently preceded ERP peaks, a cross-correlation analysis was conducted across participants which resulted in an overall best-fit lag of 4 samples (equal to 15.6ms). After correcting TRFs for this time shift, the time-dependent similarity between the ERP and TRF topographies was assessed using the spatial correlation $r_{2D}$, which can be interpreted analogously to Pearson’s correlation *r* (i.e., $r_{2D} = -1$ for perfectly inverted topographies and $r_{2D} = 1$ for perfectly identical topographies) (Murray et al. 2008). Topographic correlations were computed for each participant and both conditions from averaged waveforms in a sample-wise manner over $-$50ms to 500ms. The grand-averages across participants of the resulting waveforms are presented in Fig. 4E, which shows overall moderate to high correlations and higher post-trigger similarity between the topographies of ERPs and TRFs to attended speech with a mean correlation of $r_{2D} = 0.78$ compared to $r_{2D} = 0.60$ for ignored speech. The maximum grand-average topographic correlation of $r_{2D} = 0.89$ was observed at 136.7ms, corresponding to the ERP N1 peak latency for the attended condition. The significance of this observation was validated by a non-parametric permutation test, which identified a latency range centered at 144.5ms as having significantly higher correlations for responses to attended compared to ignored speech, together with earlier as well as later effects that will not be pursued further.

The second correlation analysis tested the ERP-TRF waveform similarity at each individual scalp site after time-lag correction by calculating Pearson’s correlation *r* between the grand-average waveforms across participants over the interval $-$50ms to 500ms. The results are depicted in Fig. 4F. Overall, correlations tended to be moderate to high at posterior scalp sites and highest at frontocentral sites. While all correlations were significant in themselves ($p < 0.001$) which provided statistical evidence in favor of working hypothesis 1 (see Fig. 1), there was a significant difference (*t*(63) = 14, $p < 0.001$), with higher correlations between ERPs and TRFs across the scalp to attended (*r* = 0.88$\pm$0.06; M$\pm$SD across channels) relative to ignored speech (*r* = 0.81$\pm$0.08; M$\pm$SD across channels).

### Auditory attention decoding performance

**Fig. 5:**
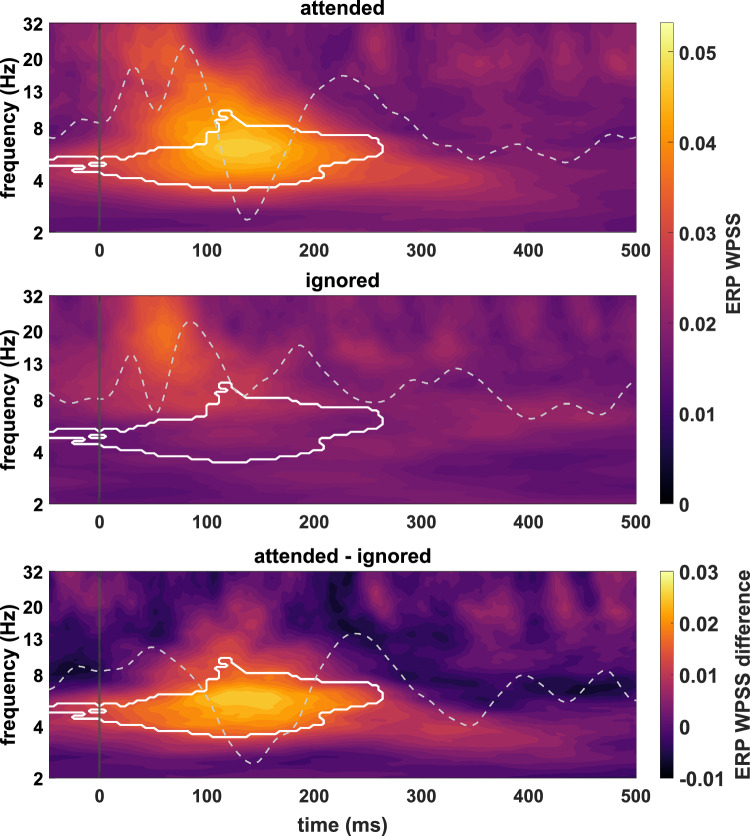
Grand-average WPSS time-frequency plots across subjects at channel Cz for ERPs to attended (top) and ignored (middle) speech as well as the corresponding difference plot (bottom). Grey dashed lines represent the grand-average speech-evoked ERPs across subjects (top and middle) and their difference wave (bottom), all centered around 0$\upmu$V with identical scaling. White outlines delimit time-frequency areas for which a non-parametric permutation test indicated significant differences between conditions. The maps show that the ERP waveform within the theta range was significantly more consistent across single sweeps when speech was attended. This attention effect extended approximately across the first 250ms of the ERPs, with the global maximum being precisely aligned with the N1 peak latency of the attended condition.

Auditory attention decoding performance was tested for ERP and TRF N1-P2 amplitudes, SR accuracy, and ERP WPSS averaged within the N1-P2 window across the theta band. Prior to averaging, the WPSS was computed for all frequencies from 2-32Hz and tested for group-level effects of attention at channel Cz. Indeed, the grand-average WPSS time-frequency plots across subjects shown in Fig. 5 show a strong phase consistency in the theta range for ERPs to attended but not to ignored speech, which is further emphasized in the difference plot. A non-parametric permutation test confirmed the statistical significance of this effect, which extended approximately across the first 250ms of the ERPs and ended shortly after the P2 peak of the grand-average ERP to attended speech. This analysis demonstrates that attending to a target stream led to a more consistent response waveform within the theta band with maximum consistency around the N1 peak latency of the attended condition.

**Fig. 6:**
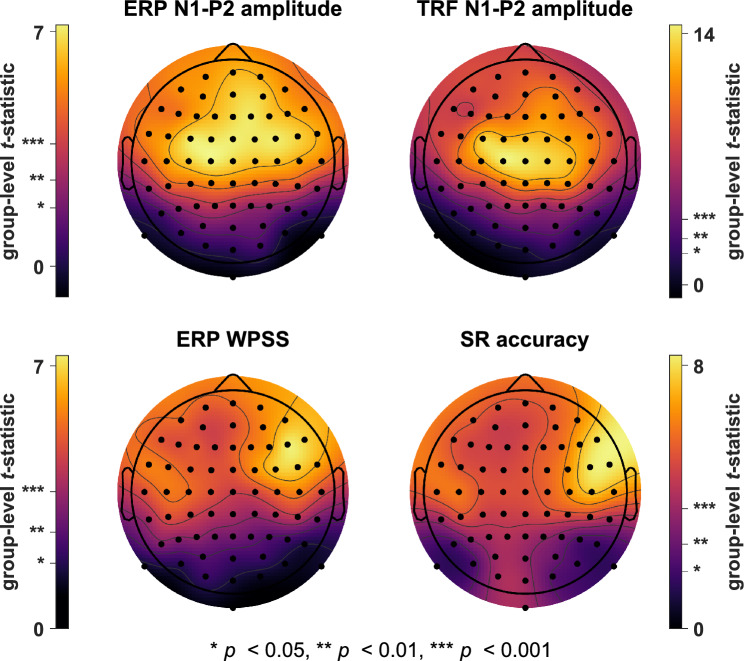
Topographic comparison of attention decoding performances between N1-P2 amplitudes extracted from speech-evoked ERPs and TRFs, ERP WPSS, and SR accuracies. Note that the topographic plots are scaled individually to highlight the patterns across the scalp. These channel-wise analyses at the group-level yielded *t*-statistic topographies with high correlations between maps for ERP and TRF N1-P2 amplitudes as well as between maps for ERP WPSS and SR accuracy.

The channel-wise *t*-statistics at the group-level for all attention decoding measures are summarized in Fig. 6 and reveal two key findings. First, all measures presented a strong separation between the attended and ignored conditions at the vast majority of channels with a strong enhancing effect of attention on all measures and overall highly significant mean *t*-statistics (ERP N1-P2 amplitude: 4.3$\pm$2.4, TRF N1-P2 amplitude: 7.8$\pm$4.0, ERP WPSS: 4.3$\pm$1.6, SR accuracy: 4.8$\pm$1.6; M$\pm$SD across channels). Second, and especially important with regard to the present working hypotheses, the single-channel analyses revealed pair-wise similarities between the topographic patterns of the ERP and TRF N1-P2 amplitude *t*-statistics on the one hand, and the ERP WPSS and SR accuracy *t*-statistics on the other. The *t*-statistic topographies for ERP and TRF N1-P2 amplitudes exhibited global maxima at midline frontocentral channels. In contrast, while the maps for SR accuracy and ERP WPSS also showed higher values over frontocentral areas, they presented lateralized distributions at frontal and frontotemporal electrodes with global maxima over the right hemisphere (electrode site F6 for ERP WPSS and FT8 for SR accuracy). A topographic correlation analysis validated the strong similarity between the *t*-statistic maps of SR accuracy and ERP WPSS ($r_{2D}$ = 0.89, $p < 0.001$) as well as between the maps of ERP and TRF N1-P2 amplitude ($r_{2D}$ = 0.95, $p < 0.001$), thereby providing strong statistical evidence in favor of working hypothesis 2 (see Fig. 1).

## Discussion

Based on the assumptions that critical speech landmarks evoke ERPs and selective auditory attention enhances the N1 component, the main objective of the present work was to gain insight into how these ERP modulations drive selective speech tracking in multi-speaker environments. To this end, speech-evoked ERPs to acoustic edges were extracted from ongoing EEG recorded during a dual-speaker selective attention task, and their relation to linear modeling techniques commonly applied to analyze the neural representation of speech was analyzed in several different ways. Taken together, these analyses revealed three key observations: First, ERPs elicited by acoustic edges in speech were strongly modulated by attention, with larger N1 components to attended compared to ignored streams. Second, forward modeling produced TRF waveforms that were remarkably accurate approximations of speech-evoked ERPs and showed equivalent attention effects. Lastly, SR backward modeling revealed effects of attention that were in line with those based on the waveform consistency of ERPs within a time-frequency area corresponding to the N1 component as quantified by phase-locking across sweeps. These results are fully consistent with our working hypotheses (see Fig. 1) and suggest that the repeated elicitation of the N1 effect through sustained allocation of attention plays a critical role in the tracking of selectively attended speech.

### Listening to speech generates auditory ERPs that are modulated by attention

Segmenting the ongoing EEG based on triggers extracted from speech dynamics revealed auditory ERPs with distinct components including a clear P1-N1-P2 complex, especially in responses to attended streams. Since the segmentation markers were derived from suprathreshold events in speech onset envelopes, this suggests a bottom-up mechanism in the generation of speech-evoked ERPs, which are triggered by physical properties inherent to speech. However, the significant N1 amplitude difference between averaged ERPs to attended and ignored speech also implicates endogenously driven top-down attention mechanisms that modulate the ERP generation process. Salient intensity modulations in speech may be considered as acoustic edges, i.e., impulse-like events similar to clicks or tone pips that are traditionally used to evoke auditory ERPs. In their seminal work, Hillyard and colleagues (Hillyard et al. 1973; Picton and Hillyard 1974) demonstrated that the effects of attention on auditory ERPs to clicks and tones are manifested in the N1 effect; that is, selectively attending a stream of sounds while ignoring another leads to an enhanced N1 component in response to the former. The present results emphasize the importance and generality of this mechanism by suggesting that the same N1 effect holds for speech-evoked ERPs if extracted from appropriate speech representations. Similar attentional modulations may also be expected during selective listening paradigms using different types of continuous auditory stimuli such as music or environmental sounds, which would be a fruitful area for future research.

### Speech-evoked ERPs and TRFs represent the same brain response when obtained from the same speech representation

In line with our first working hypothesis, the present results confirm that if forward modeling of speech tracking is conducted with the same speech representation used as regressor as that used for ERP trigger extraction, speech-evoked ERPs and resulting TRFs represent the same brain response. While there are some minor discrepancies between the observed ERPs and the derived TRFs, these can be attributed to two main factors. First, TRFs consistently preceded ERPs by a best-fit lag of 15.6ms. This discrepancy appears to be a consequence of the implemented ERP extraction method, which was based on statistical properties of speech onset envelopes, namely the sample standard deviation. In comparison to the linear regression approach that essentially performs a precise deconvolution, trigger extraction for ERPs relied on an empirically chosen threshold, which may have influenced the resulting ERP onset and peak latencies. By adjusting the envelope threshold accordingly, ERP and TRF components could in principle be aligned accurately. However, since the focus here was on response morphologies rather than absolute latencies, and the chosen parameters yielded strong ERP-TRF similarities, the standard threshold of twice the standard deviation was chosen as the default. Second, ERP and TRF waveforms and between-condition amplitude differences varied slightly outside of the P1-N1-P2 time interval. While this could be influenced by the envelope threshold as well, the regularization applied during ridge regression may be the dominant factor. Since regularization constrains TRF weights by penalizing large coefficients to prevent overfitting, the resulting TRFs may lose details and become more generalized.

The one-to-one correspondence between speech-evoked ERPs and the modeled TRFs was strongly confirmed by the correlation analyses, which provided objective evidence for highly similar topographies and waveforms across channels. In a similar vein, comparable observations have previously been made when comparing standard click-evoked auditory brainstem responses to their TRF representations modeled to speech (Maddox and Lee 2018) and tone-evoked cortical auditory ERPs to their TRF counterparts (Reetzke et al. 2021). By demonstrating a direct link between ERPs and TRFs, the present findings provide strong support for the hypothesis that TRFs may be interpreted as reflecting true brain responses to various kinds of continuous auditory stimulation (Ding and Simon 2013; Crosse et al. 2015; Di Liberto et al. 2015; Broderick et al. 2018; Verschueren et al. 2021; Kaufman and Zion-Golumbic 2023). As an alternative to the forward modeling approach, the impulse response of the brain to a continuous stimulus can also be obtained by performing a cross-correlation between M/EEG and stimulus and the resulting cross-correlation function closely resembles the corresponding TRF (Crosse et al. 2016). Therefore, the same principle also appears to hold for cross-correlation analyses that have studied cortical responses to speech (Hertrich et al. 2011; Hambrook and Tata 2014; Schmitt et al. 2022).

### SNR enhancement produced by selective auditory attention and the N1 attention effect improves TRF and SR modeling in multi-speaker scenarios

The importance of SNR improvement in favor of cortical activity related to selectively attended versus ignored speech, largely due to the N1 effect, becomes apparent in many analyses, including TRF as well as SR modeling.

Overall, TRF modeling produced highly accurate estimations of speech-evoked ERPs as confirmed by the ERP-TRF waveform correlations, with highest similarities at frontocentral channels and a symmetrical pattern across hemispheres, which corresponds to the typical frontocentral topographies of late auditory ERP components including the N1 (Picton et al. 1974). The ERP-TRF waveform correlation analysis revealed that TRFs represented significantly better estimations of ERPs for attended compared to ignored speech, emphasizing that selective attention had enhanced the SNR. This was also supported by the time-resolved topographic ERP-TRF correlation analysis, which revealed more similar topographies for responses to attended speech and reached significance during several time windows, most prominently in the N1 interval. Similar conclusions can also be drawn from the TRF and ERP attention decoding analysis, which contrasted N1-P2 amplitudes between conditions of attention. The comparison across channels revealed highly correlated topographies for group-level *t*-statistics which provides further evidence for a direct ERP-TRF correspondence. Importantly, best separability between conditions was found at anterior regions where the N1 component was largest; the distributions showed *t*-statistic maxima at frontocentral channels and minima towards posterior channels. Despite the similar topographies, the mean *t*-statistic across channels for TRFs was noticeably larger than for ERPs (7.8 vs. 4.3). This potentially demonstrates that the positive effect of consistently enhanced ERP components on SNR is amplified during TRF estimation, perhaps because ERPs with clear and consistent deflections may be modeled especially well and ERPs with subdued components proportionally worse, which in turn would increase the separability between TRFs to attended and ignored speech.

Consistent with our second working hypothesis, the attention decoding *t*-statistic scalp maps for the SR accuracy and the waveform consistency (WPSS) of speech-evoked ERP N1-P2 responses in the theta band yielded highly similar topographies. Although decoding performances were again biased towards anterior scalp areas, as observed for N1-P2 amplitudes in ERPs and TRFs, their overall scalp distribution differed considerably. In contrast to the frontocentral distribution of the N1-P2 amplitudes, the separability between conditions for the SR accuracy and the N1-P2 phase consistency was greatest over the anterior region of the temporal lobe in the right hemisphere. This distribution is congruent with the asymmetric sampling in time (AST) hypothesis (Poeppel 2003), which suggests a preferred sampling of speech in time windows corresponding to gamma periods in the left hemisphere ($\sim$20-40ms, phonetic information) and windows matching theta cycles in the right hemisphere ($\sim$150-250ms, syllabic information). In accord with this proposal, previous research has identified a general bias for the right hemisphere to be dominant in the synchronization of M/EEG theta band activity to spoken sentences (Luo and Poeppel 2007; Ding and Simon 2012b), which has been similarly demonstrated in studies on selective attention to speech (Vander Ghinst et al. 2016; Puschmann et al. 2017), sound envelope tracking (Abrams et al. 2008; Chalas et al. 2022), and attention decoding (Kerlin et al. 2010). Overall, the present findings suggest that the consistent generation of an enhanced N1 component in auditory ERPs to acoustic edges in selectively attended speech improves the SNR between goal-related and background cortical activity. By doing so, it possibly enhances the accuracy of SR modeling and leads to better attention decoding performance in multi-speaker scenarios. These effects are also manifested in the phase consistency of EEG theta band activity, as discussed below.

### Acoustic edges and the N1 component may contribute to modulations of theta activity in neural tracking of speech

There are two main competing theories regarding how brain activity synchronizes to auditory input; the first involves an active alignment of ongoing neural oscillations to the stimuli, and the second a superposition of additional neural activity evoked by the stimuli (Aiken and Picton 2008; Lakatos et al. 2013; Ding and Simon 2014; Kayser et al. 2015; Zoefel et al. 2018; Zuk et al. 2021). The former proposal suggests that low frequency neural oscillations phase-align to auditory stimuli in order to enhance stimulus processing through optimized excitability (Lakatos et al. 2005; Luo and Poeppel 2007; Schroeder et al. 2010; Lakatos et al. 2013; Doelling et al. 2014), a mechanism that has been proposed to rely on a syllable-based theta alignment when considering speech (Luo and Poeppel 2007). In contrast, the syllable-theta correspondence might also be established by the latter mechanism, with the addition of evoked N1 activity in the theta band. Theta speech tracking has been related to speech clarity (Etard and Reichenbach 2019) and intelligibility (Luo and Poeppel 2007; Howard and Poeppel 2010; Ghitza 2012). Similarly, two speech features that have been found to be important for high intelligibility are syllabic information (Ghitza 2012) and acoustic edges (Howard and Poeppel 2010), which are proposed to be correlated while they exhibit similar occurence rates within speech that approximately match the theta band (Ding and Simon 2014; Oganian et al. 2023); this observation also accords with the AST hypothesis (Poeppel 2003). In the present study, an EEG segmentation method based on acoustic edges in speech (i.e., potentially syllabic information) revealed the presence of speech-evoked ERPs similar to those reported in recent studies (Oganian et al. 2023) including consistent P1-N1-P2 complexes. Importantly, the manifestation of the N1 component was correlated with phase-locked theta activity (see also Klimesch et al. 2004; Trenado et al. 2009; Low and Strauss 2011; Corona-Strauss and Strauss 2017), which resulted in an enhanced representation of speech within the theta band, closing the loop and allowing an interpretation of theta speech tracking in terms of an additive evoked response mechanism.

It is important to note that any definitive statement regarding the extent to which the two proposed mechanisms discussed above contribute to the neural synchronization to speech would require definite knowledge about the generative origin of ERPs (Sayers et al. 1974; Makeig et al. 2002; Fell et al. 2004; Mäkinen et al. 2005; David et al. 2006; Hanslmayr et al. 2007; Min et al. 2007; Sauseng et al. 2007; Mishra et al. 2012; Burgess 2012); a question that is not within the scope of this work. However, the present findings provide explanations for some of the effects that have been observed for theta speech tracking that may be based on the typical behavior of ERPs, e.g., for the relation of theta activity to speech intelligibility. Previous studies have found that modifying speech to be unintelligible by means of speech-noise chimeras (Luo and Poeppel 2007) or an increase of background noise floor (Etard and Reichenbach 2019) impairs speech tracking through theta activity. These modifications can be thought of as a masking of acoustic edges and syllabic information, which has been shown to diminish the signal quality of auditory ERPs to syllables by smearing component amplitudes and latencies (Billings et al. 2013). Translating this effect to the aforementioned studies on speech tracking, the diminished speech-evoked ERPs due to masking would consequently impair the cortical representation of speech by effectively reducing the SNR with respect to background activity. Due to the absence or smearing of P1-N1-P2 complexes, this effect would additionally express itself through the collapse of theta speech tracking.

In summary, it is clear that bottom-up, stimulus-driven mechanisms have to be complemented by top-down attentional processes to achieve successful neural tracking of speech. Focusing on the situation of selectively attended speech in a multi-speaker environment, the present findings indicate that the auditory N1 effect (Hillyard et al. 1973; Picton and Hillyard 1974) improves the SNR in favor of the target speech compared to its competitors. The generation of ERPs with enhanced N1 components could then provide reliable cues for higher-order cortical networks to lock on to attended information, ultimately facilitating the tracking and processing of the desired speech stream. These observations and the underlying methods can be directly translated to several use cases. With respect to scientific research that investigates how the brain synchronizes to selectively attended speech, the present work suggests that standard ERP processing techniques such as the analysis of phase stability (WPSS) provide valuable alternatives to the commonly applied TRF and SR approaches. Comparing the results of different analysis methods could thereby help to evaluate the robustness of any observed effects or reveal additional similarities as well as potential differences between the outcomes of traditional ERP and linear modeling approaches. From a technological perspective, the emergence of smart technologies such as neuro-steered hearing aids (Geirnaert et al. 2021) implies a need for stable attention decoding strategies that can operate online while keeping the computational cost low, for which the presented ERP amplitude and phase analyses represent promising candidates. Additionally, the proposed strategies may also be integrated in clinical settings where they could serve as rapid approaches for evaluating patients’ attention and language comprehension in situations of comprehending natural speech. In these scenarios, the absence of attention effects on N1 amplitude or phase stability could be related to deficits that may be worth examining more thoroughly.
